# Functional relationship between woody plants and insect communities in response to *Bursaphelenchus xylophilus* infestation in the Three Gorges Reservoir region

**DOI:** 10.1002/ece3.7716

**Published:** 2021-06-03

**Authors:** Zhuang Wang, Lijuan Zhao, Jiaqi Liu, Yajie Yang, Juan Shi, Junbao Wen, Ruihe Gao

**Affiliations:** ^1^ Beijing Key Laboratory for Forest Pest Control College of Forestry Beijing Forestry University Beijing China; ^2^ College of Marxism Shanxi Agricultural University Taigu China; ^3^ College of Forestry Shanxi Agricultural University Taigu China; ^4^ Shanxi Dangerous Forest Pest Inspection and Identification Center Taigu Shanxi China

**Keywords:** insect functional group, pine wilt disease, plant community structure, redundancy analysis

## Abstract

To study the effect of the invasion of *Bursaphelenchus xylophilus* on the functional relationship between woody plants and insect communities, the populations of tree species and insect communities were investigative in the Masson pine forests with different infestation durations of *B. xylophilus*. In this study, the number of *Pinus massoniana* began to decrease sharply, whereas the total number of other tree species in the arboreal layer increased gradually with the infestation duration of *B. xylophilus*. The principal component analysis ordination biplot shows that there was a significant change in the spatial distribution of woody plant species in different Masson pine forest stands. Additionally, a total of 7,188 insect specimens were obtained. The insect population showed an upward trend in stand types with the increase of pine wilt disease infection periods, which demonstrated that the insect community had been significantly affected by the invasion of *B. xylophilus*. However, the insect diversity indexes were not significantly different among Masson pine forest stands. The structure of insect functional groups changed from herbivorous (He) > omnivorous (Om) > predatory (Pr) > parasitic (Pa) > detritivorous (De) in the control stand to He > Pa > Om, De > Pr after *B. xylophilus* infestation in the forests. The results showed that the populations of He, Pa, and De increased after the invasion of *B. xylophilus*, but the populations of Pr decreased. Moreover, the redundancy analysis ordination biplots reflected the complicated functional relationship between woody plant communities and insects after the invasion of *B. xylophilus*. The present study provides insights into the changes in the community structure of woody plants and insects, as well as the functional relationship between woody plant communities and insect communities after invasion of *B. xylophilus*.

## Graphical abstract

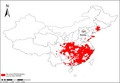

## 1 INTRODUCTION

With increase in the frequency of international trade, some invasive alien species are directly or indirectly introduced into new forest ecosystems. As an external disturbance factor, they inevitably affect the productivity, nutrient cycle, hydrological system, species diversity, and information transmission of the new ecosystem (Ding et al., 2008; Lovett et al., 2006; Westphal et al., 2008). The plant community structure and composition of the forest ecosystem can be significantly changed, and the succession rate of the community can be accelerated after the infestation of invasive alien species (Castello et al., 1995; Gao et al., 2015; Spiegel & Leege, 2013). Consequently, the species composition and population structure of the animals, insects, and microorganisms that depend on the plant community may also undergo considerable changes (Karban, 2011; Li et al., 2016; Visakorpi et al., 2019).

**FIGURE 1:**
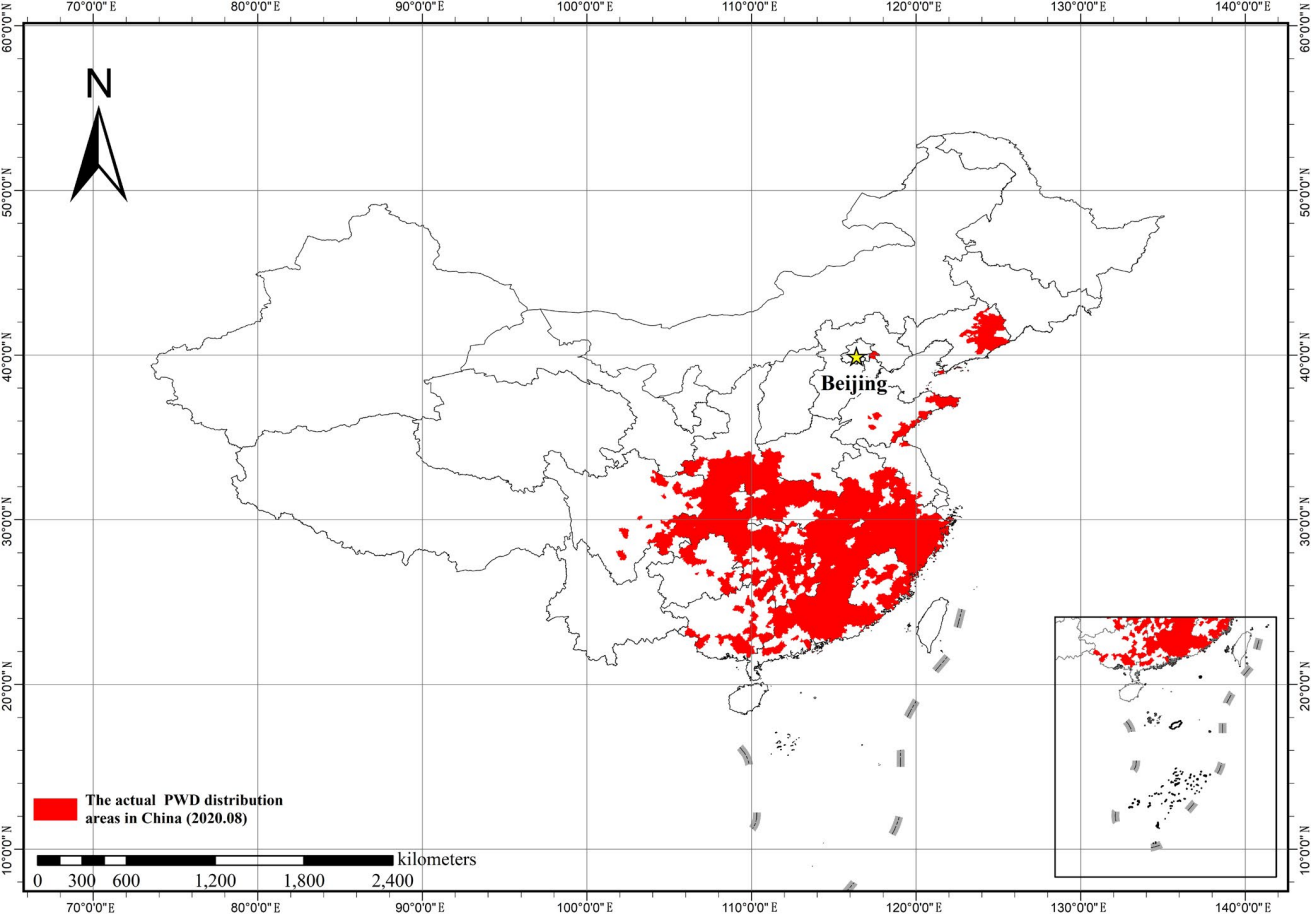
The actual epidemic distribution areas of pine wilt disease in China in August 2020. (Data obtained from the No. 4 and No. 18 bulletin of National Forestry and Grassland Administration in 2020, 2020)

As one of the most dangerous and destructive forest noxious organism, *Bursaphelenchus xylophilus* (Steiner and Buhrer) Nickle (Nematoda: Aphelenchoididae) causes the pine wilt disease (PWD) and has a strong destructive effect on pine forest ecosystems in China, Japan, Korean, Spain, and Portugal (Abelleira et al., 2011; Futai, 2013; Sousa et al., 2011; Zhao, 2008). The pine sawyer beetle, *Monochamus alternatus,* is the most efficient insect vector for spreading *B. xylophilus* in China and other East Asia counties (Li et al., 2020; Mamiya & Enda, 1972; Zhao, 2008). The average number of *B. xylophilus* carried by *M. alternatus* is 18,000 per beetle, and the maximum number is 289,000 (Zhao, 2008). In China, *B. xylophilus* was first discovered in 40 black pines in September 1982 at Sun Yat‐Sen's Mausoleum in Nanjing (Cheng et al., 1983; Wan et al., 2005). Since then, the degree of damage inflicted by *B. xylophilus* is a continuous large‐scale occurrence, which has caused huge economic and ecological losses around China (Shi et al., 2013; Wan et al., 2005; Zhao, 2008). By August 2020, PWD had spread to 18 provinces and 666 counties in China. In addition, the extent of this disease in China has displayed explosive growth in the past two years, with 283 and 85 new counties affected with PWD epidemic added in 2018 and 2019, respectively. Moreover, the PWD outbreaks have spread to areas where the annual average temperature is below 10°C. In China, the western infection area of *B. xylophilus* has reached Xichang City, Sichuan Province, and the northern part has colonized Kaiyuan City, Liaoning Province, directly threatening the safety of pine forest resources in northern China (Figure 1).

Previously published works have documented that plant succession is a major factor that affects the insect community's composition and structure (June et al., 2006; Siemann, 1998). As an important environmental indicator, insect species are usually linked to plant communities and are widespread in forest ecosystems (Cédric et al., 2013; Siemann, 1998; Vandewalle et al., 2010). Woody plant species provide much of the habitat and resource base for insects (Brown et al., 2001; Trotter et al., 2008). A greater diversity of plants provides a greater diversity of resources for insects; consequently, the increase in plant species richness also leads to an increase in insect species richness (Haddad et al., 2001; Knops et al., 1999). Furthermore, a higher diversity of herbivorous insects can provide more food sources for insect predators and parasitoids (Knops et al., 1999).

For the dominant tree species with a large biogeographic distribution, changes in the communities and population structure of this species are likely to affect many other tree or animal species at large landscape scales (Trotter et al., 2008). Masson pine is an important pine tree species in the Three Gorges Reservoir area, which is widely distributed from the bank of the Yangtze River to the top of the mountain (Gao et al., 2015). In this area, the main coniferous forest type was the Masson pine pure forest before the invasion of *B. xylophilus* in 2006. In the following years, this epidemic spread rapidly, killing Masson pine trees occupying a large area.

To date, little is known about the functional relationship between woody plants and insect communities in response to *B. xylophilus* infestation. To address this issue, the survey of woody plant and insect communities was conducted in the eastern part of the Three Gorges Reservoir region of China. The aim of this work was to address the following questions: after the invasion of *B. xylophilus* (i) how do woody plant species change? (ii) What impact does the change in woody plant structure have on the insect community structure and composition? and (iii) how is the functional relationship between the woody plant community and insects impacted?

## 2 MATERIALS AND METHODS

### 2.1 Study sites

This study was carried out in Yiling District (latitude 30°32′–31°28′N, longitude 110°51′–111°39′E), which is located in the eastern part of the Three Gorges Reservoir region and contained the demarcation point of the upper and middle reaches of the Yangtze River (Gao et al., 2019). In this area, PWD was first detected on Masson pine in 2006. Local forestry authorities removed all infected Masson pine trees in 2012; from then, the coniferous forest regenerate naturally. The research was conducted on five Masson pine stand types, which were classified based on the duration of PWD infection from 2006 to 2012 (Table 1). Additionally, each Masson pine stand type had three repeated stands, and there were three permanent 15 m × 15 m Masson pine plots in each stand.

**TABLE 1:** Stand characteristics of five Masson pine stands infected by *B. xylophilus*

Stand Type	Duration of PWD infection up to 2012/Year	Elevation/m	Slope/。	Number of stems/ha^−1^	Mean DBH/cm
ST1	0	125.00 ± 6.39	21.00 ± 2.78	2,144 ± 205.58	7.93 ± 1.26
		110.67 ± 7.95	25.00 ± 3.24	1622 ± 174.62	7.54 ± 1.41
		188.67 ± 4.37	26.67 ± 2.15	1,800 ± 265.11	9.60 ± 2.42
ST2	1	172.33 ± 7.32	17.33 ± 4.16	1567 ± 226.90	8.50 ± 1.51
		294.33 ± 4.96	23.00 ± 3.78	1556 ± 271.05	8.67 ± 1.77
		169.00 ± 3.90	15.67 ± 4.63	1,288 ± 219.06	10.94 ± 2.12
ST3	3	234.33 ± 3.20	17.33 ± 7.76	1,267 ± 289.17	9.62 ± 2.72
		201.00 ± 10.39	32.33 ± 6.33	1,400 ± 243.77	10.21 ± 3.42
		208.00 ± 4.52	21.67 ± 4.38	1,245 ± 155.56	8.57 ± 2.26
ST4	5	197.67 ± 7.89	15.00 ± 5.57	1,211 ± 281.38	13.33 ± 3.45
		278.00 ± 9.30	22.67 ± 4.25	1,222 ± 162.49	10.39 ± 2.27
		220.00 ± 14.42	26.67 ± 625	1,134 ± 145.01	7.11 ± 2.10
ST5	7	217.33 ± 9.84	17.33 ± 4.51	1,011 ± 170.03	11.42 ± 2.35
		186.00 ± 12.39	13.33 ± 5.78	967 ± 203.77	11.32 ± 1.99
		267.00 ± 4.39	22.33 ± 2.65	911 ± 172.86	13.73 ± 2.93

### 2.2 Field surveys

From July to August 2012, the woody plants in the arboreal layer were investigated in each of the 45 plots. For each plot, the basic environmental factors, including elevation, slope, and canopy density, were recorded. The woody plants with a diameter at breast height (DBH) ≥ 2.5 cm were investigated using the method of “Tally” which includes species name, height, DBH, and crown.

### 2.3 Insect sampling and specimen identification

For each plot, insect sampling was conducted once every 7 days from June to August in 2013 and 2014. Insect specimens were collected by means of “sweep net sampling,” “Malaise trap sampling,” and “window trap sampling.” For the “sweep net sampling” method, a muslin insect swept net was used for sweeping in each corner of the 45 plots, for a total of more than 200 times net was swept for each plot. For the “Malaise trap sampling,” a Malaise trap was placed in the center of the 45 plots and was mainly used to trap small insects. For this method, the collected insect specimens could be directly stored in 75% alcohol, which ensured the necessary quality for subsequent molecular identification. Additionally, five window traps (29.5 cm × 19.0 cm × 0.2 cm) were placed at the center and the four end points according to the “five‐point sampling” method. The window traps were suspended at a height of approximately 3 m, and the collection device was filled with 75% alcohol in order to preserve the collected insect species.

According to relevant professional books and references, insect individuals were identified as exactly as possible to genus and species. The sampled insect specimens were divided into five groups: herbivorous insects (He), predatory insects (Pr), parasitic insects (Pa), omnivorous insects (Om), and detritivorous insects (De). Moreover, the dominant insect group was the one whose individual number accounted for more than 10% of the total collected insects, common groups accounted for 1.0%–10% of the total number, and the rare groups accounted for less than 1% of the total number (Wang & Wang, 2010).

### 2.4 Data analysis

One‐way ANOVA and Fisher's least significant difference (LSD) test with an alpha value of *p* <.05 were used to compare the changes in woody plant and insect community structure. The indexes of richness (S), Margalef's richness (H), Shannon's diversity (H’), Simpson's dominance (D_2_), Simpson's evenness (E), and Pielou's evenness (J) were calculated to compare the variation of insect diversity among different Masson pine forest stands (Table 2). Principal component analysis (PCA) was conducted using CANOCO 5.0 (Microcomputer Power, Ithaca, NY, USA) to determine the plant community structure in different Masson pine stands. In CANOCO 5.0, redundancy analysis (RDA) based on a constrained linear model was used to analyze the ordination relation of woody plant species and insect species. All statistical analyses were performed using GraphPad Prism 6.0 (GraphPad Software, La Jolla, CA, USA) and SPSS 22.0 for Windows (SPSS Inc., Chicago, IL, USA).

**TABLE 2:** The formulas used to calculate insect diversity indexes of five Masson pine stands infected by *B. xylophilus*

Indexes	Formula
Richness (S)	Number of species
Margalef's richness (H)	(S−1)/ln(*N*)
Shannon's diversity (H′)	‐∑*P_i_* ln(*P_i_*)
Simpson's dominance (D_2_)	1/∑*P_i_^2^*
Simpson's evenness (E)	D_2_/S
Pielou's evenness (J)	H′/ln(S)

Note

*N* is the number of insect individuals in different plots; *P_i_* is the proportion of individuals belong to species. Formulas from Shannon (1948), Simpson (1949), Margalef (1958), and Pielou (1966).

## 3 RESULTS

### 3.1 Changes in woody plant community structure

The total number of *P. massoniana* in the healthy ecosystem (ST1) was higher than that in the infected stands (Table 3). With the increase in the infestation duration of PWD, the number of *P. massoniana* decreased sharply, and the statistical difference between different stand types reached a significant level (*F* = 6.99, *p* < .01). The total number of other tree species in the arboreal layer (except *P. massoniana*) increased slowly with the increase in the degree of PWD infestation. Additionally, the difference in the total number of the remaining species, ST4 and ST5, reached a significant level when compared with the healthy Masson pine ecosystem (*p* < .05).

**TABLE 3:** The number of stems for all tree species measured in five Masson pine forest sites infected for different periods by pine wood nematode

Stand types	*P. massoniana*	Other spp.
ST1	1,303 ± 174.62a	533 ± 65.11a
ST2	933 ± 171.05ab	556 ± 117.06ab
ST3	704 ± 243.77bc	600 ± 155.56a
ST4	537 ± 162.49bc	726 ± 145.01b
ST5	348 ± 203.77c	793 ± 72.86b

Note

Values are mean ± *SD* of three replicates for each stand type. For each column, values with different letters are significantly different at *p* =.05.

**FIGURE 2:**
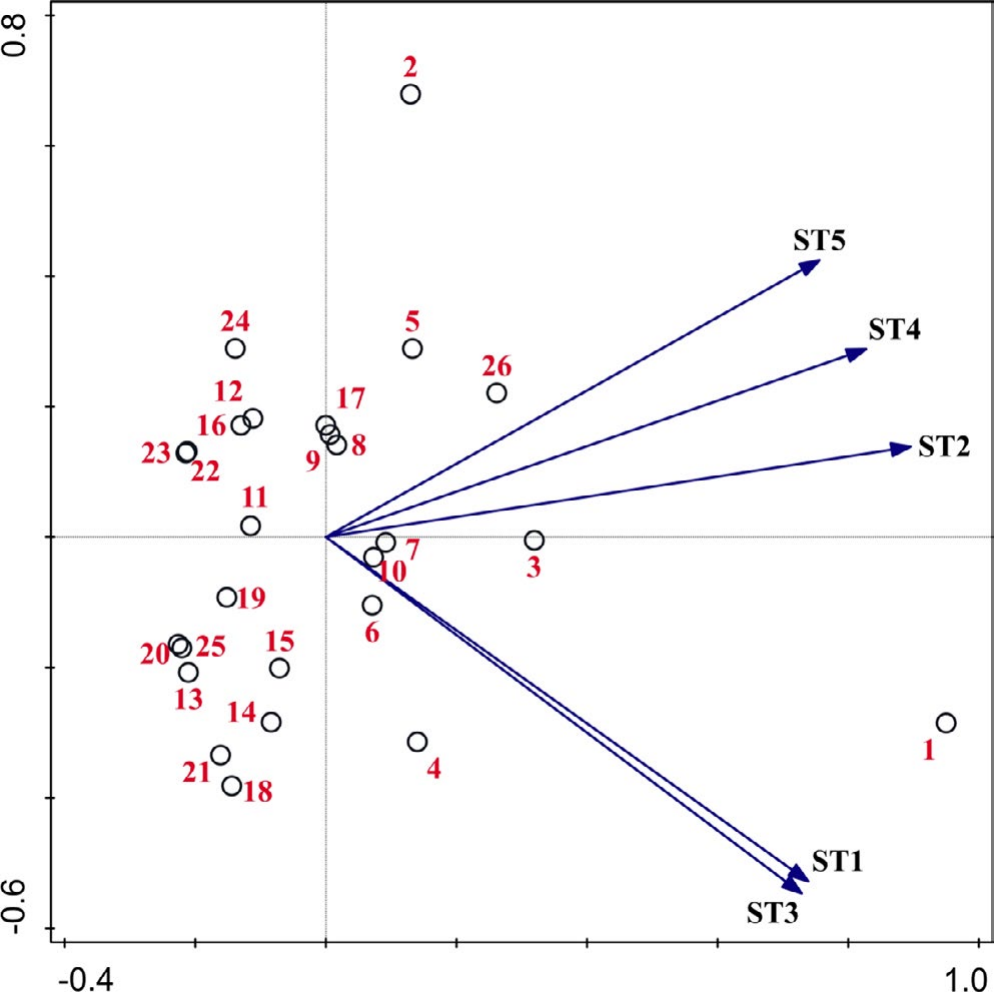
Principal component analysis ordination diagram of plant community structure in different Masson pine forest stands. 1. *Pinus massoniana*; 2. *Cinnamomum camphora*; 3. *Quercus aliena*; 4. *Quercus variabilis*; 5. *Loropetalum chinensis*; 6. *Rhus chinensis*; 7. *Celtis bungeana*; 8. *Trachycarpus fortunei*; 9. *Cotinus coggygria*; 10. *Litsea cubeba*; 11. *Symplocos paniculata*; 12. *Rhus typhina*; 13. *Dalbergia hupeana*; 14. *Ilex cornuta*; 15. *Albizia kalkora*; 16. *Symplocos caudata*; 17. *Aralia chinensis*; 18. *Rhamnus parvifolius*; 19. *Castanea mollissima*; 20. *Pistacia chinensis*; 21. *Deutzia grandiflora*; 22. *Camellia oleifera*; 23. *Melia azedarach*; 24. *Sapium sebiferum*; 25. *Sabina chinensis*; 26. Remaining species

The PCA result and ordination biplot of woody plant species in different Masson pine forest stands are shown in Table 4 and Figure 2. *P. massoniana* was the most important tree in the tree layer in ST1, with an importance value of up to 67.23%. With the increase in the duration of *B. xylophilus* infestation, the importance value of *P. massoniana* in the corresponding stand types decreased sharply to 53.61%, 50.24%, 42.65%, 29.24% in ST2‐5. On the contrary, the importance value of *Cinnamomum camphora*, *Quercus aliena*, *Quercus variabilis*, and *Loropetalum chinensis* showed a rapid upward trend. After 7 years of continuous *B. xylophilus* infestation, *C. camphora* had surpassed *P. massoniana* to become the most important tree species in the tree layer in ST5.

**TABLE 4:** The PCA result of woody plant species in different Masson pine forest stands

Canonical axes	Eigenvalues	Cumulative percentage
Axis 1	0.6267	62.67
Axis 2	0.1695	79.62
Axis 3	0.1155	91.17
Axis 4	0.0542	96.58

### 3.2 Insect community structure

In this study, we collected and identified 7,188 individual insects, representing 510 species from 15 orders and 152 families (Table 5). There were significant differences in the levels of order, family, and individual among the collected insects. Overall, the dominant insect communities were Hemiptera, Coleoptera, Hymenoptera, Diptera, and Lepidoptera, which made up more than 10% of the total at the levels of family, species, and individuals. Other insect communities accounted for a relatively small proportion of the total insect population.

**TABLE 5:** Composition of insect communities in Masson pine forest

Order	Family		Species		Individual	
	Number	Percentage/%	Number	Percentage/%	Number	Percentage/%
Hemiptera	23	15.13	74	14.51	1693	23.55
Coleoptera	32	21.05	144	28.24	1682	23.40
Hymenoptera	26	17.11	88	17.25	1,279	17.79
Diptera	22	14.47	57	11.18	945	13.15
Lepidoptera	19	12.50	81	15.88	857	11.92
Orthoptera	11	7.24	32	6.27	337	4.69
Ephemeroptera	2	1.32	3	0.59	92	1.28
Blattaria	4	2.63	7	1.37	69	0.96
Neuroptera	3	1.97	6	1.18	68	0.95
Odonata	4	2.63	7	1.37	66	0.92
Mantodea	1	0.66	3	0.59	46	0.64
Thysanoptera	1	0.66	3	0.59	24	0.33
Megaloptera	2	1.32	2	0.39	20	0.28
Dermaptera	1	0.66	2	0.39	5	0.07
Plecoptera	1	0.66	1	0.20	5	0.07
Total	152	100	510	100	7,188	100

There was an upward trend in the insect population at the levels of order, family, species, and individual among different stand types (Table 6). However, there was little variation in insect community composition at the order, family, and species levels among different stand types, and the difference between them was not significant. Conversely, the individuals were significantly different among different stand types (*F* = 204.20, *p* < .01), and the difference between ST1‐2 and ST3‐5 was significant.

**TABLE 6:** The composition of insects in different Masson pine forest stand types

Stand types	Order	Family	Species	Individuals
ST1	11.33 ± 0.58a	61.67 ± 4.04a	93 ± 11.53a	318 ± 61.55a
ST2	10.33 ± 0.58a	63.33 ± 7.57a	91.67 ± 5.13a	414 ± 42.44ab
ST3	10 ± 2.65a	66 ± 17.69a	104.33 ± 31.47a	546 ± 100.68c
ST4	12 ± 2.65a	66.67 ± 12.58a	105 ± 31.95a	522.67 ± 26.00c
ST5	10.67 ± 1.15a	64 ± 5.29a	104 ± 15.59a	595.33 ± 34.65c

Note

Values are mean ± *SD* of three replicates for each stand type. For each column, values with different letters are significantly different at *p* =.05.

Overall, the orders of Hemiptera, Coleoptera, Hymenoptera, and Diptera were the dominant insect groups in different Masson pine forest stand types, and each of their relative abundance was all greater than 10% (Table 7). The dominant insect groups in Hemiptera were Cicadellidae (17.42%), Aphidoidea (17.07%), and Pentatomidae (15.36%). The dominant insect groups in Coleoptera were Scarabaeidae (19.08%). The dominant insect groups in Hymenoptera were Formicidae (37.29%), Ichneumonidae (14.62%), and Vespidae (11.26%). The dominant insect groups in Diptera were Tipulidae (33.91%) and Cecidomyiidae (14.92%).

**TABLE 7:** Composition of insect communities collected from five Masson pine forest stand types

Order	ST1			ST2			ST3			ST4			ST5		
	Number of species	Number of individuals	Number of abundance/%	Number of species	Number of individuals	Number of abundance/%	Number of species	Number of individuals	Number of abundance/%	Number of species	Number of individuals	Number of abundance/%	Number of species	Number of individuals	Number of abundance/%
Hemiptera	12.67 ± 3.06A	44.67 ± 12.74a	14.05	14.33 ± 1.15A	134.00 ± 31.48a	32.37	14.67 ± 3.21A	119.33 ± 20.50a	21.86	17.67 ± 9.29A	133.33 ± 53.26a	25.51	18.33 ± 7.09A	133.00 ± 103.94a	22.34
Coleoptera	31.00 ± 1.00A	116.67 ± 8.33a	36.69	25.33 ± 7.37A	88.67 ± 36.12a	21.42	23.00 ± 9.54A	107.00 ± 66.30a	19.60	31.00 ± 11.27A	111.67 ± 36.56a	21.36	31.33 ± 6.81A	136.67 ± 49.52a	22.96
Hymenoptera	14.67 ± 4.62AB	58.67 ± 22.85a	18.45	12.67 ± 3.21A	54.67 ± 27.79a	13.20	24.33 ± 9.29B	92.33 ± 22.03a	16.91	12.67 ± 3.79A	71.67 ± 5.13a	13.71	17.00 ± 5.57AB	149.00 ± 18.36b	25.03
Diptera	9.67 ± 2.08A	34.67 ± 12.86a	10.90	11.67 ± 6.03A	51.33 ± 15.63ab	12.40	13.67 ± 3.51A	95.33 ± 10.02b	17.46	10.00 ± 1.73A	70.33 ± 60.35ab	13.46	9.67 ± 3.21A	63.33 ± 17.56ab	10.64
Lepidoptera	8.00 ± 1.73A	16.00 ± 3.61a	5.03	15.00 ± 7.00A	42.33 ± 30.11ab	10.23	16.67 ± 3.06A	71.67 ± 20.65b	13.13	17.33 ± 9.07A	85.33 ± 28.71b	16.33	16.33 ± 9.45A	70.33 ± 38.53b	11.81
Orthoptera	5.33 ± 0.58AB	21.00 ± 10.44a	6.60	6.00 ± 1.00AB	18.33 ± 6.66a	4.43	5.33 ± 1.53AB	31.33 ± 14.01a	5.74	7.67 ± 2.89B	25.33 ± 13.87a	4.85	4.00 ± 1.00A	16.33 ± 10.01a	2.74
Ephemeroptera	1.00 ± 0.00A	1.67 ± 1.55a	0.52	1.67 ± 1.15A	9.00 ± 3.61a	2.17	1.00 ± 1.00A	7.00 ± 6.08b	1.28	1.33 ± 0.58A	5.67 ± 7.23b	1.08	1.67 ± 1.15A	7.33 ± 8.50b	1.23
Blattaria	2.00 ± 0.00B	5.00 ± 2.65a	1.57	1.00 ± 0.00AB	5.00 ± 3.61a	1.21	0.67 ± 1.15A	1.00 ± 1.73a	0.18	1.67 ± 0.58AB	5.67 ± 3.51a	1.08	2.00 ± 0.00B	6.33 ± 2.31a	1.06
Neuroptera	2.33 ± 0.58A	4.67 ± 4.62a	1.47	1.33 ± 0.58A	3.33 ± 2.31a	0.81	1.00 ± 1.00A	4.33 ± 3.79a	0.79	1.67 ± 0.58A	4.33 ± 2.08a	0.83	1.67 ± 0.58A	6.00 ± 1.73a	1.01
Odonata	3.67 ± 2.31A	11.00 ± 8.54a	3.46	1.33 ± 2.31A	4.00 ± 6.93a	0.97	0.67 ± 1.15A	3.00 ± 5.20a	0.55	1.00 ± 1.00A	2.67 ± 2.52a	0.51	0.67 ± 1.15A	2.33 ± 2.08a	0.22
Mantodea	2.33 ± 0.58B	3.00 ± 1.73a	0.94	1.00 ± 1.00AB	2.00 ± 2.00a	0.48	1.33 ± 1.15AB	6.67 ± 6.51a	1.22	1.00 ± 1.00AB	2.00 ± 2.00a	0.38	0.33 ± 0.58A	1.67 ± 2.89a	0.28
Thysanoptera	0.33 ± 0.58AB	1.00 ± 1.73a	0.31	0	0	0	1.33 ± 0.58B	4.33 ± 1.53b	0.79	0.67 ± 0.58AB	1.33 ± 1.53ab	0.26	0.33 ± 0.58AB	1.33 ± 2.31ab	0.22
Megaloptera	0	0	0	0.67 ± 1.15A	1.33 ± 2.31a	0.32	0.67 ± 1.15A	2.67 ± 4.62a	0.49	0.67 ± 1.15A	1.67 ± 2.89a	0.32	0.33 ± 0.58A	1 ± 1.73a	0.17
Dermaptera	0	0	0	0	0	0	0	0	0	0.67 ± 0.58B	1.67 ± 2.08a	0.32	0	0	0
Plecoptera	0	0	0	0	0	0	0	0	0	0	0	0	0.33 ± 0.58	1.67 ± 2.89	0.28
Total	93.00 ± 11.53A	318.00 ± 61.55a	100.00	91.67 ± 5.13A	414.00 ± 42.44ab	100.00	104.33 ± 31.47A	546.00 ± 100.68c	100.00	105.00 ± 31.95A	522.67 ± 26.00c	100.00	104.00 ± 15.59A	595.33 ± 34.65c	100.00

Note

Values are mean ± *SD* of three replicates for each stand type. Mean values of the number of species within a row followed by different uppercase letters are significantly different at *p* =.05 level, mean values of the number of individuals within a row followed by different lowercase letters are significantly different at *p* =.05 level.

**FIGURE 3:**
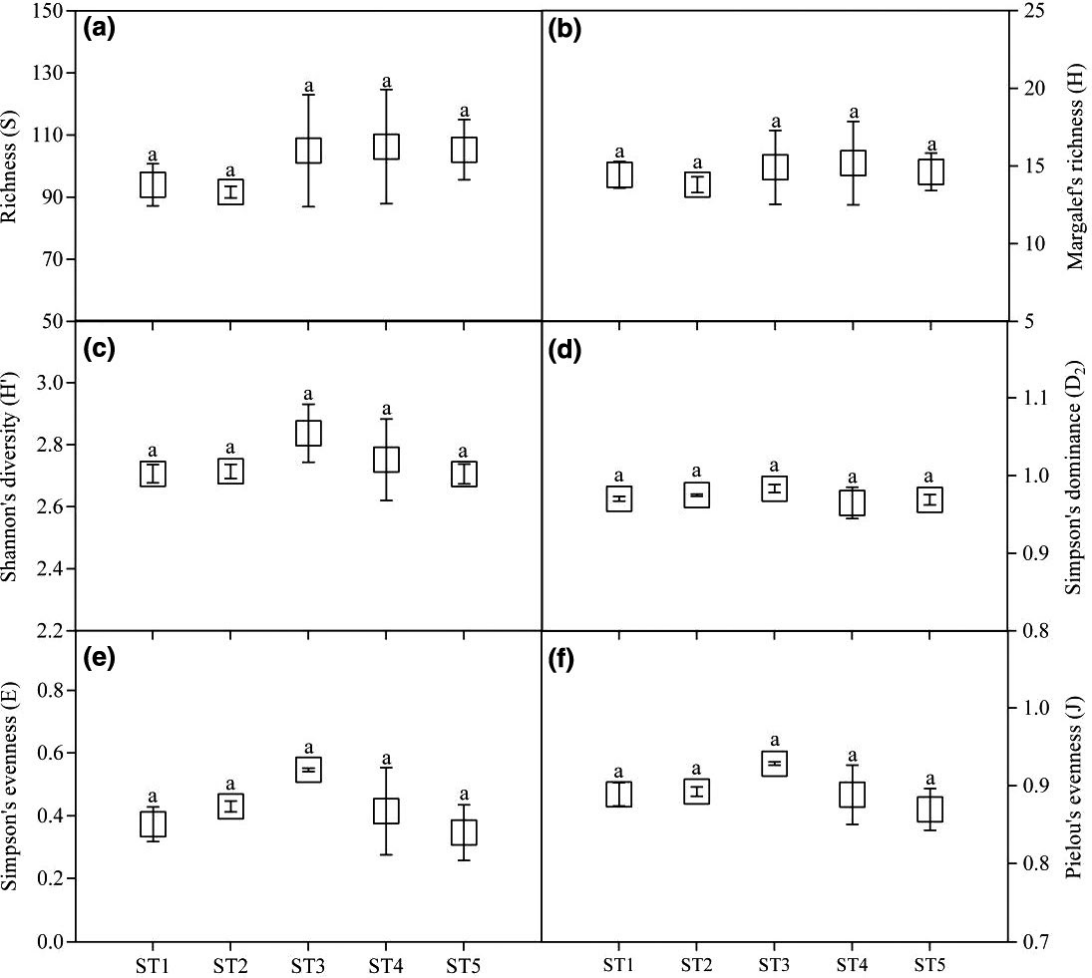
The variation of insect diversity indexes among five Masson pine forest stands infected by *B. xylophilus*. (a) the difference of richness (S); (b) the difference of Margalef's richness (H); (c) the difference of Shannon's diversity (H′); (d) the difference of Simpson's dominance (D_2_); (e) the difference of Simpson's evenness (E); (f) the difference of Pielou's evenness (J)

As revealed by Figure 3, the insect diversity indexes were not significantly different among five Masson pine forest stands infected by *B. xylophilus*. Compared with the control stands, the S and H were slightly decreased in ST2 and then showed an increasing trend over the course of PWD infection (Figure 3a and Figure 3b). The changes of H′, D_2_, E, and J were fluctuated, but no significant change trends were observed among different stand types.

### 3.3 Insect functional groups

The structure of insect functional groups had changed over the course of *B. xylophilus* infestation (Table 8). The magnitude of relative abundance for insect functional groups in the control stand type (ST1) was He > Om > Pr > Pa > De, and after infection, the magnitude of the relative abundance for insect functional groups changed (ST2‐ST5: He > Pa > Om, De > Pr). After the invasion of *B. xylophilus*, each of the relative abundance of He, Pa, and De was increased by varying degrees, with the values of Pa and De increasing from 7.86% to 16.69% and from 2.10% to 11.16%, respectively. Meanwhile, the values of the relative abundance of Pr and Om showed a downward trend.

**TABLE 8:** The relative abundance of insect functional groups in five Masson pine forest stand types infected for different periods of time by *B. xylophilus*

Functional groups	Relative abundance (%)				
	ST1	ST2	ST3	ST4	ST5
He	58.49	69.81	64.16	63.9	58.96
Pa	7.86	9.66	13.06	6.76	16.69
Pr	14.78	8.7	8.91	10.01	6.66
Om	16.77	8.7	9.04	8.16	10.69
De	2.1	3.14	4.82	11.16	7

Abbreviations: De, detritivorous insects; He, herbivorous insects; Om, omnivorous insects; Pa, parasitic insects; Pr, predatory insects.

**FIGURE 4:**
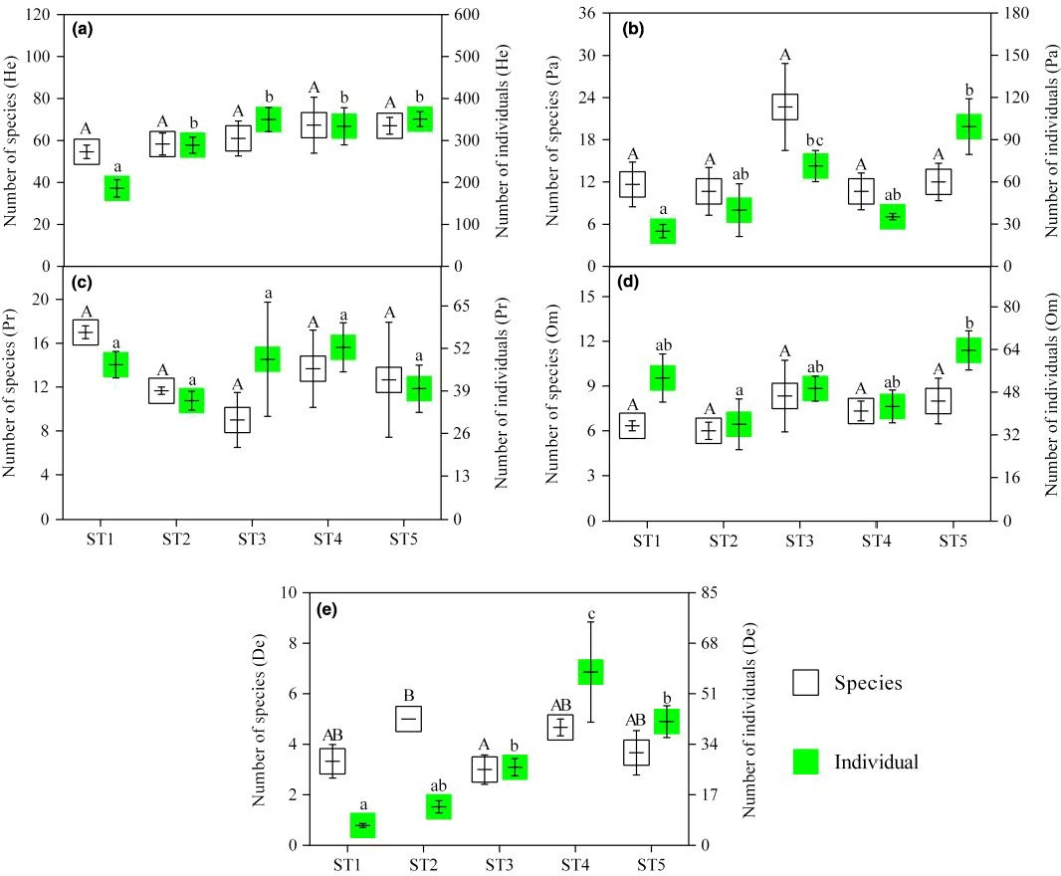
The difference of insect functional groups at the level of species and individuals in different Masson pine forest stands. (a) the difference of insect functional groups for herbivorous insects; (b) the difference of insect functional groups for parasitic insects; (c) the difference of insect functional groups for predatory insects; (d) the difference of insect functional groups for omnivorous insects; (e) the difference of insect functional groups for detritivorous insects. Mean values of the number of species among different stand types followed by different uppercase letters are significantly different at *p* =.05 level, mean values of the number of individuals among different stand types followed by different lowercase letters are significantly different at *p* =.05 level

Figure 3 shows the difference in insect functional groups at the levels of species and individuals in different Masson pine forest stands. In terms of He, the number of insect species and individuals increased with the increase in invasion duration of *B. xylophilus*, and the difference in individuals among different stand types reached a significant level (Figure 4a). For Pa and De, the number of individuals showed an increasing trend, but there was no obvious change pattern as to the number of species (Figure 4b and e). There was little variation in the species and individuals of Pr among different stand types (Figure 4c). After the Masson pine forest infested by *B. xylophilus*, the individual number of Om showed an increasing trend, but there was little difference in the number of Om species among different stand types. (Figure 4d).

**FIGURE 5:**
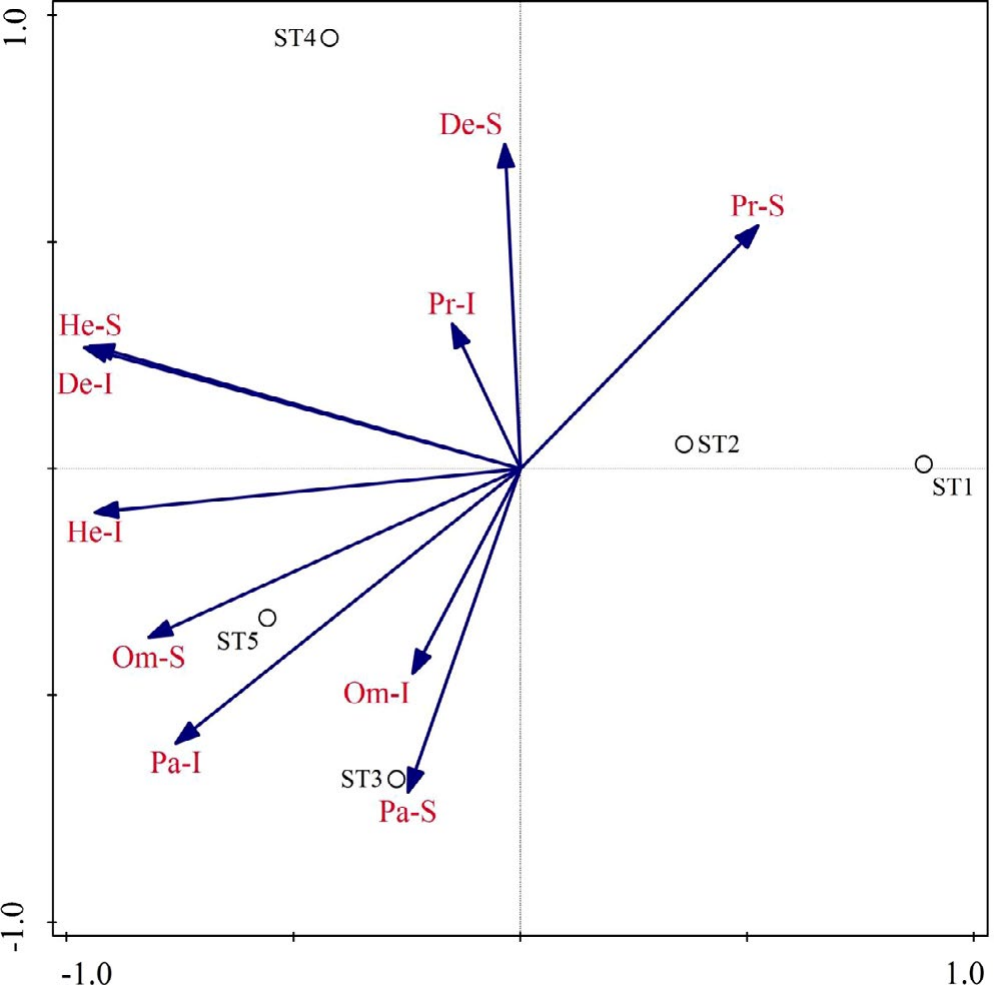
Principal component analysis ordination diagram of insect functional groups in different Masson pine forest stands. He‐S: number of species of herbivorous insects; He‐I: number of individuals of herbivorous insects; Pa‐S: number of species of parasitic insects; Pa‐I: number of individuals of parasitic insects; Pr‐S: number of species of predatory insects; Pr‐I: number of individuals of predatory insects; Om‐S: number of species of omnivorous insects; Om‐I: number of individuals of omnivorous insects; De‐S: number of species of detritivorous insects; De‐I: number of individuals of detritivorous insects

The PCA result and ordination biplot of insect functional groups in different Masson pine forest stands are shown in Table 9 and Figure 5, which reflects the distribution of the insect community in each stand type. Pr‐S was positively associated with ST1 and ST2 but was negatively associated with ST3‐ST5. The most important insect functional groups in ST3 and ST5 were Pa‐S, Pa‐I, Om‐S, Om‐I, and He‐I. However, the He‐S, De‐S, De‐I, and Pr‐I groups played essential roles in ST4.

**TABLE 9:** The PCA result of insect functional groups variables in different Masson pine forest stands

Canonical axes	Eigenvalues	Cumulative percentage
Axis 1	0.6929	69.29
Axis 2	0.1878	88.07
Axis 3	0.0641	94.48
Axis 4	0.0552	100

### 3.4 Functional relationship between woody plants and insects

For a more intuitive understanding of the complicated functional relationship between woody plant community and insect functional groups, a redundancy analysis was conducted using 16 woody plant species with an importance value of over 1, and five insect functional groups with the numbers of species and individuals. As shown by the RDA results in Table 10, a total of 66.9% canonical eigenvalues in insect functional groups can be explained through ordination by the selected woody plant species.

**TABLE 10:** The summary of statistical of the redundancy analysis

Canonical axes	Eigenvalues	Cumulative explained variation (%)	Cumulative explained fitted variation (%)	Sum of all eigenvalues	Sum of all canonical eigenvalues
RDA1	0.434	43.35	90.44	1	0.669
RDA2	0.368	80.19	97.88		
RDA3	0.133	93.50	99.85		
RDA4	0.065	100	100		

**FIGURE 6:**
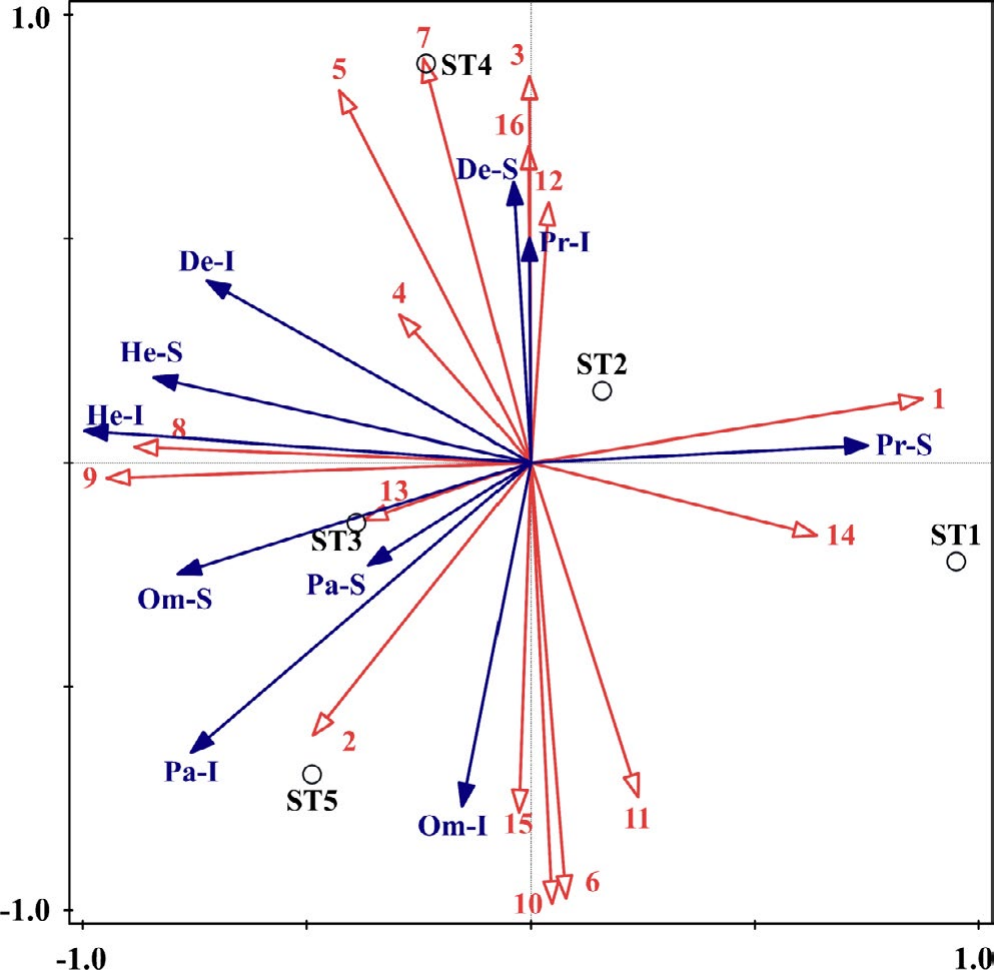
Results of the RDA ordination biplot presenting woody plant species and insect functional groups in different Masson pine forest stands. For woody plant species variables: 1. *Pinus massoniana*; 2. *Cinnamomum camphora*; 3. *Quercus aliena*; 4. *Quercus variabilis*; 5. *Loropetalum chinensis*; 6. *Rhus chinensis*; 7. *Celtis bungeana*; 8. *Trachycarpus fortunei*; 9. *Cotinus coggygria*; 10. *Litsea cubeba*; 11. *Symplocos paniculata*; 12. *Rhus typhina*; 13. *Dalbergia hupeana*; 14. *Ilex cornuta*; 15. *Albizia kalkora*; 16. *Symplocos caudata*. For insect functional group variables: He‐S: Species of herbivorous insects; He‐I: individuals of herbivorous insects; Pa‐S: species of parasitic insects; Pa‐I: individuals of parasitic insects; Pr‐S: species of predatory insects; Pr‐I: individuals of predatory insects; Om‐S: species of omnivorous insects; Om‐I: individuals of omnivorous insects; De‐S: species of detritivorous insects; De‐I: individuals of detritivorous insects

The RDA ordination biplot with woody plant species and insect functional groups along the first two axes is presented in Figure 6. As revealed by the ordination biplot, there is a gradient in plots along axis 1, from ST1 very positive to ST5 negative; however, they are pushed in different directions along axis 2. Moreover, Pr‐S was positively associated with the distribution of *P. massoniana* and *Ilex cornuta* in ST1 and ST2. De‐S and Pr‐I were positively associated with the growth of some broad‐leaved tree species, including *Q. aliena*, *Q. variabilis*, *Loropetalum chinensis*, *Celtis bungeana*, *Rhus typhina*, and *Symplocos caudatahe*. The distribution of *Trachycarpus fortune*, *Cotinus coggygria*, and *Dalbergia hupeana* may promote an increase in the numbers of He‐S, He‐I, Om‐S, and De‐I. Moreover, there was a significant negative correlation between parasitic insects (Pa‐S and Pa‐I) and the distribution of *P. massoniana*, but the parasitic insects were positively associated with some broad‐leaved tree species, including *C. camphora*, *T. fortune*, *C. coggygria*, *D. hupeana*, *Rhus chinensis*, *Litsea cubeba*, *Symplocos paniculate*, and *Albizia kalkora*.

## 4 DISCUSSION

The present study provides new insights into the functional relationship between woody plants and insect communities after the invasion of *B. xylophilus*. This study mainly examined the community structure and abundance of woody plants and insects in response to *B. xylophilus* infestation in the eastern part of the Three Gorges Reservoir region of China. The research was conducted on five Masson pine stand plots, which were classified based on the duration of PWD infection from 2006 to 2012. Local forestry authorities removed all infected Masson pine trees in 2012, after which the Masson pine stands regenerated naturally.

Under natural conditions, *B. xylophilus* infestation will cause serious damage and loss to a healthy pine forest ecosystem, but with the continuous succession and natural recovery of the ecosystem, the species diversity of the plant community will increase significantly, and the pine forest will evolve and develop in a more advanced direction (Shi et al., 2013; Spiegel & Leege, 2013; Zhao, 2008). The results of this study show that the invasion of *B. xylophilus* can affect the structure composition of woody plants and significantly affect the spatial and structural distribution of plant species in the arboreal layer. Specifically, the number of stems for all woody tree species measured in five Masson pine forest plots was infected for different periods by *B. xylophilus*. With the increase in the infestation duration of *B. xylophilus*, the number of *P. massoniana* began to decrease sharply. Meanwhile, the total number of other tree species in the arboreal layer (except *P. massoniana*) increased gradually with an increase in the degree of *B. xylophilus* infestation in the ecosystem. Similar results were found in many previous studies, which showed that the invasion of *B. xylophilus* can lead to the succession of the pure *P. massoniana* forest ecosystem into a mixed coniferous and broadleaf forest ecosystem or even a broadleaf forest ecosystem (Gao et al., 2015; Shi et al., 2007; Wang et al., 2014). By comparing and analyzing the niche indices of the main woody plant species, the vegetation communities in the woody plant layer did not degenerate in the direction of the shrub layer after the invasion of the Masson pine ecosystem by *B. xylophilus*. In general, the structure of new forest ecosystems formed by succession tends to be more stable and has a strong ability to resist forest pests and diseases (Hambäck et al., 2000; Humphrey et al., 1999; Jobidon et al., 2004; Li et al., 2012).

To study the effect of the invasion of *B. xylophilus* on insects, insect communities were sampled from five Masson pine stand plots with different durations of *B. xylophilus* infection. In this study, a total of 7,188 individual insects was collected and identified, representing 510 species from 15 orders and 152 families. Overall, the orders of Hemiptera, Coleoptera, Hymenoptera, and Diptera were the dominant insect groups in different Masson pine forest stand types, each with a relative abundance greater than 10%. This finding is consistent with that of a previous study in which the insects of the dominant orders belonged to Coleoptera, Hymenoptera, and Diptera in a coniferous forest ecosystem affected by *B. xylophilus* in Zhejiang Province (Gao et al., 2013). Additionally, the number of insect species and individuals showed an upward trend in Masson pine forest stand types with increasing PWD infection periods, which demonstrated that the insect community was significantly affected by the invasion of *B. xylophilus*. Previous studies have indicated that a greater diversity of woody plant species provides much more habitats and resources for insects (Brown et al., 2001; Cédric et al., 2013; Root, 1973; Siemann, 1998; Trotter et al., 2008; Vandewalle et al., 2010). Therefore, through this study, we found that the invasion of *B. xylophilus* can affect the community structure and composition, change the spatial and structural distribution of woody plant species, and further affect the distribution of insect community structure and individual populations.

As a multidimensional property of natural system, biodiversity represents the variety and heterogeneity of organisms and usually quantified by diversity indices (Daly et al., 2018; Morris et al., 2014). Insect diversity is an important part of biodiversity and one of the key indicators to study the structure and function of insect community (Barton & Evans, 2017; Christensen et al., 1996). In this research, we compared insect diversity indexes of S, H, H', D_2_, E, and J among five Masson pine forest stands infected by *B. xylophilus*. However, the insect diversity indexes were not significantly different among stands. After the invasion of *B. xylophilus*, the values of S and H were slightly decreased in ST2 and then showed an increasing trend over the course of PWD infection. These results indicate that outbreaks of PWD increase S and H of insect species, which may occur due to the emergence of abundant woody plants after cutting infected trees in the Masson pine ecosystem (Gao et al., 2015; Li & Shao, 2006). However, there is no obvious change trend of the values of H′, D_2_, E, and J. The variation of these results may be due to the selected plots only infected by *B. xylophilus* 1–7 years, which was too soon for insect species to adequately respond to changes in forest composition where infected Masson pine trees have severely declined (Gao et al., 2015; Kim et al., 2010; Spiegel & Leege, 2013). As a whole, the variation of insect diversity indexes is not yet well understood and requires further study.

The structure of insect functional groups can reflect the development and health status of a specific forest ecosystem (Haddad et al., 2001; June et al., 2006; Karban, 2011; Visakorpi et al., 2019). In this study, the magnitude of relative abundance for insect functional groups in the control stand type (ST1) was He > Om > Pr > Pa > De, whereas after infection, the magnitude of relative abundance for insect functional groups was altered (ST2‐ST5: He > Pa > Om, De > Pr). This result indicated that the structure of insect functional groups changed over the course of *B. xylophilus* infestation in the Masson pine forests. As a crucial part of the biological communities in forest ecosystems, the population dynamics and spatial distribution of insect functional groups are significantly restricted by woody plant species (Bezemer et al., 2014; Cédric et al., 2013; Root, 1973; Tchakonté et al., 2015; Vandewalle et al., 2010). Additionally, the outbreak of PWD also has multiple effects on growth rates, canopy structure, and composition of woody plant species, which in turn may alter the structure of insect functional groups (Gao et al., 2015; Li et al., 2012; Root, 1973; Veblen et al., 1991). Moreover, the number of insect species and individuals of herbivorous insects increased with an increase in the invasion duration of *B. xylophilus*, and the difference of individuals reached a significant level among stand types. In order to control the population of herbivorous insects and enhance degradation of plant and insect residues (Haddad et al., 2001; June et al., 2006; Trotter et al., 2008; Visakorpi et al., 2019), the relative abundance of parasitic insects and detritivorous insects in this study also increased by varying degrees after the invasion of *B. xylophilus*, with values increasing from 7.86% to 16.69% and from 2.10% to 11.16%, respectively.

As revealed by the RDA ordination graph, the population spatial distribution of insect functional groups was significantly restricted by woody plant species. The correlation between parasitic insects and woody plant species indicated that broad‐leaved tree species may provide more resources and ecological niches for parasitic insects. This result is consistent with a previous study showing that the number and diversity index of parasitic insects increased with an increase in the proportion of broad‐leaved tree species in a coniferous forest ecosystem in Zhejiang Province (Wang et al., 2014). According to “Resource Concentration Hypothesis,” the increase in plant species diversity can provide more habitats and food resources, which may significantly affect the structure of insect functional groups and increase the species and individual numbers of herbivorous and parasitic insects (Gao et al.,^2013^, ^2018^; Knops et al., 1999; Root, 1973; Taki et al., 2010). As the population of parasitic and predatory insects continues to increase, the number of herbivores, including *M. alternatus*, which is the vector of *B. xylophilus*, may be limited.

## 5 CONCLUSIONS

The invasion of *B. xylophilus* can affect the composition of woody plant communities and change the spatial and structural distribution of woody plant species. The structure of insect functional groups changed from He > Om > Pr > Pa > De in the control stand to He > Pa > Om, De > Pr over the course of *B. xylophilus* infestation in the Masson pine forests. Moreover, the redundancy analysis ordination biplots reflected the complicated functional relationship between the woody plant community and insects after the invasion of *B. xylophilus*, and the invasion of *B. xylophilus* can promote the population growth of herbivorous insects, parasitic insects, and detritivorous insects.

## CONFLICT OF INTEREST

The authors have no conflicts of interest to declare.

## AUTHOR CONTRIBUTION

**Zhuang Wang:** Conceptualization (equal); Data curation (equal); Writing‐original draft (equal); Writing‐review & editing (equal). **Lijuan Zhao:** Conceptualization (equal); Data curation (equal); Writing‐original draft (equal); Writing‐review & editing (equal). **Jiaqi Liu:** Formal analysis (equal); Software (equal). **Yajie Yang:** Formal analysis (equal); Software (equal). **Juan Shi:** Writing‐review & editing (equal). **Junbao Wen:** Funding acquisition (equal); Writing‐review & editing (equal). **Ruihe Gao:** Conceptualization (equal); Funding acquisition (equal); Writing‐original draft (equal); Writing‐review & editing (equal).

## Data Availability

The data are available in the Dryad database under the following link: https://doi.org/10.5061/dryad.c59zw3r6d
